# 

**Published:** 1904-04-23

**Authors:** 


### NOTICE TO CORRESPONDENTS.

All MSS., Letters, Books for Review, and other matters intended for the Editor should be addressed The Editor, "The Hospital," The Hospital Buildings, 28 & 29 Southampton Street, Strand, W.C.

The Editor cannot undertake to return rejected MS., even when accompanied by stamped directed envelope.

### Notice to Advertisers and Subscribers.

All Advertisements, Orders for Copies of The Hospital, and Business Communications should be addressed to **THE MANAGER (Not to the Editor)**, The Hospital, 28 & 29 Southampton Street, Strand, London, W.C.

The Cost of Subscription, post free, is as follows:—Per week, 3½d.; per quarter, 3s. 9d.; per half-year, 7s. 6d.; per annum, 15s. Foreign and Colonial:—Per annum, 19s. 6d., payable, in all cases, in advance.

**NOTICE.—Vol. XXXV. of "The Hospital" (October 1903 to March 1904), in handsome cloth binding, will shortly be on sale at the office of this Journal, price 10s. 6d. Cases for binding the Numbers contained in this Volume, 2s. Index to Vol. XXXV., 3½d., post free.**

**The Monthly part for April is now ready. Price 1s., by post, 1s. 3d.**

### THE STUDENT OF MEDICINE.

### APPOINTMENTS.

C. C. Bullmore, L.R.C.P.&S.Edin., L.F.P.S.Glasg., District and Workhouse Medical Officer of the Falmouth Union. **W. T. Freeman**, M.D.Durh., F.R.C.S., L.R.C.P.Lond., Senior Assistant Physician to the Royal Berkshire Hospital. **T. H. Goodman**, M.R.C.S.Eng., L.S.A., District Medical Officer of the Risbridge Union. **P. E. Hill**, M.R.C.S., L.S.A., Medical Officer of Health for the Crickhowell Combined Districts. **S. H. Johnson**, M.R.C.S., L.R.C.P.Lond., Medical Officer of Health for the Sculcoates Rural District. **W. L. McCormac**, M.B., Ch.B.Vict., Resident Assistant Medical Officer at the Leeds Union Infirmary. **F. Heurtt Oliver**, L.R.C.P.Lond., L.S.A.Lond., Surgeon-Accoucheur to the City of London Lying-in-Hospital. **C. A. H. Thomson**, M.B., B.C.Cantab., Medical Officer at Falmouth, Jamaica. **J. R. H. Walker**, M.D.Edin., Medical Officer of the Risbridge Union Workhouse. **J. S. Yule**, M.D., M.S.Melb., Clinical Assistant to the Chelsea Hospital for Women.

### "The Hospital" Institutional Library.

"Hospitals and Asylums of the World." 4 Vols., with a Portfolio of Plans, complete £12 12s.
"Burdett's Hospitals and Charities, 1903." 5s. net.
"Burdett's Official Nursing Directory, 1903." 3s. net.
"Cottage Hospitals, General, Fever, and Convalescent." 10s. 6d.
"Uniform System of Accounts for Hospitals and Public Institutions." New Edition. 4s. net.
"Hospital Expenditure: The Commissariat." 2s. 6d.
"A Legal Handbook for the Use of Hospital Authorities." 2s. 6d.
"The Cottage Hospital Case Book or Register of Patients." 10s. and 12s. 6d.
All these are published by the Scientific Press, Ltd., and may be obtained through any bookseller, or direct from the publishers, 28 and 29 Southampton Street, Strand, London, W.C.

### HANDBOOKS FOR NURSES ON SPECIAL SUBJECTS.

### Ophthalmic Nursing.

By SYDNEY STEPHENSON, M.B., F.R.C.S.E.

New and Revised Edition, Illustrated, cloth, **3/6** net; **3/10** post free.

### Nursing in Diseases of the Throat, Nose, and Ear.

By P. MacLEOD YEARSLEY, F.R.C.S., M.R.C.S.

Illustrated, cloth, **2/6** post free.

### Art of Massage.

By A. CREIGHTON HALE.

Second Edition, Profusely Illustrated, **6/-** post free.

### Medical Gymnastics,

### Including the Schott (Nauheim) Movements.

By AXEL. V. GRAFSTROM, M.D.

Illustrated, cloth, **2/6** post free.

### Mental Nursing.

By WILLIAM HARDING, M.D. Ed.

Cloth, **1/-** post free.

### Fevers and Infectious Diseases:

### Their Nursing and Practical Management.

By WILLIAM HARDING, M.D. Ed.

Cloth, **1/-** post free.

*COMPLETE CATALOGUE POST FREE ON APPLICATION.*

*Of all Booksellers, or of*

THE SCIENTIFIC PRESS, LTD., 28 & 29 Southampton Street, Strand, London, W.C.

